# Non-classical 1p36 deletion in a patient with Duane retraction syndrome: case report and literature review

**DOI:** 10.1186/s13039-020-00510-5

**Published:** 2020-09-07

**Authors:** Emiy Yokoyama, Camilo E. Villarroel, Sinhué Diaz, Victoria Del Castillo, Patricia Pérez-Vera, Consuelo Salas, Samuel Gómez, Reneé Barreda, Bertha Molina, Sara Frias

**Affiliations:** 1 Departamento de Genética Humana, Instituto Nacional de Pediatría, Mexico City, Mexico; 2 Enlace Científico, Shire Pharmaceuticals México, Mexico City, Mexico; 3 Laboratorio de Genética y Cáncer, Departamento de Genética Humana, Instituto Nacional de Pediatría, Mexico City, Mexico; 4 CRIT Chiapas, Tuxtla, Mexico; 5 Laboratorio de Citogenética, Departamento de Genética Humana, Instituto Nacional de Pediatría, Mexico City, Mexico; 6 Instituto de Investigaciones Biomédicas, Universidad Nacional Autónoma de México, Avenida IMAN No. 1, Torre de Investigación, Insurgentes Cuicuilco, Coyoacán, 04530 Mexico City, Mexico

**Keywords:** 1p36 deletion, *HES3* gene haploinsufficiency, Duane retraction syndrome

## Abstract

**Background:**

Monosomy of 1p36 is considered the most common terminal microdeletion syndrome. It is characterized by intellectual disability, growth retardation, seizures, congenital anomalies, and distinctive facial features that are absent when the deletion is proximal, beyond the 1p36.32 region. In patients with proximal deletions, little is known about the associated phenotype, since only a few cases have been reported in the literature. Ocular manifestations in patients with classical 1p36 monosomy are frequent and include strabismus, myopia, hypermetropia, and nystagmus. However, as of today only one patient with 1p36 deletion and Duane retraction syndrome (DRS) has been reported.

**Case presentation:**

We describe a patient with intellectual disability, facial dysmorphism, and bilateral Duane retraction syndrome (DRS) type 1. Array CGH showed a 7.2 Mb de novo deletion from 1p36.31 to 1p36.21.

**Discussion:**

Our patient displayed DRS, which is not part of the classical phenotype and is not a common clinical feature in 1p36 deletion syndrome; we hypothesized that this could be associated with the overlapping deletion between the distal and proximal 1p36 regions. DRS is one of the Congenital Cranial Dysinnervation Disorders, and a genetic basis for the syndrome has been extensively reported. The *HES3* gene is located at 1p36.31 and could be associated with oculomotor alterations, including DRS, since this gene is involved in the development of the 3rd cranial nerve and the 6th cranial nerve’s nucleus. We propose that oculomotor anomalies, including DRS, could be related to proximal 1p36 deletion, warranting a detailed ophthalmologic evaluation of these patients.

## Background

1p36 monosomy is the most common terminal microdeletion syndrome, with an incidence of 1 in every 5000 to 10,000 newborns and is found in more than 1.2% of patients with idiopathic intellectual disability [1]. Although the diagnosis may be suspected clinically, it is often confirmed after the application of molecular cytogenetic tests. Most cases are de novo with a microdeletion size of approximately 5 Mb, ranging from 1.5 to 10.5 Mb [2, 3]. The classical phenotype is associated with distal deletion of the most terminal chromosomal band (1p36.3) and includes intellectual disability, growth retardation, microcephaly, a distinctive craniofacial dysmorphism, and other variable congenital malformations. Larger deletions extending up to 1p36.31 show a severe neurological phenotype including profound disability, epilepsy, and deafness [3]. By comparison, proximal deletions beyond 1p36.23 (usually interstitial) show non-classical features including severe intellectual disability, hirsutism, abnormal ears, coarse facies, congenital heart disease, and variable cardiomyopathy [4, 5]. These proximal deletions are more infrequent than the distal deletions, so knowledge about their associated phenotype (sometimes referred as “proximal 1p36 deletion syndrome”) is limited [4]. The common ocular manifestations or functional visual problems described in both distal and proximal deletions are strabismus (30–35%), myopia (17%), hypermetropia (67%), and nystagmus (13%) [1, 2]. The presence of Duane retraction syndrome (DRS) has only been reported on one occasion, by Neal in 2006 [6].

DRS is a congenital eye movement disorder characterized by the variable limitation of abduction and globe retraction with narrowing of the palpebral fissure on abduction [7]. Although most cases are sporadic, sometimes this disorder is part of a recognizable genetic entity, whether as a main feature or as a rare associated finding [7]. DRS has also been associated with several cytogenetic anomalies, such as deletions on chromosomes 4 and 8 or the presence of an extra marker chromosome derived from chromosome 22 [8]. Here, we describe a patient with a 1p36.21p36.31 deletion with a non-classical phenotype by reason of the uncommon DRS type 1 clinical feature. We also analyze the genes that might be implicated in the phenotype, and we propose that oculomotor anomalies including DRS, could be related to proximal 1p36 deletion.

## Case presentation

**Fig. 1:**
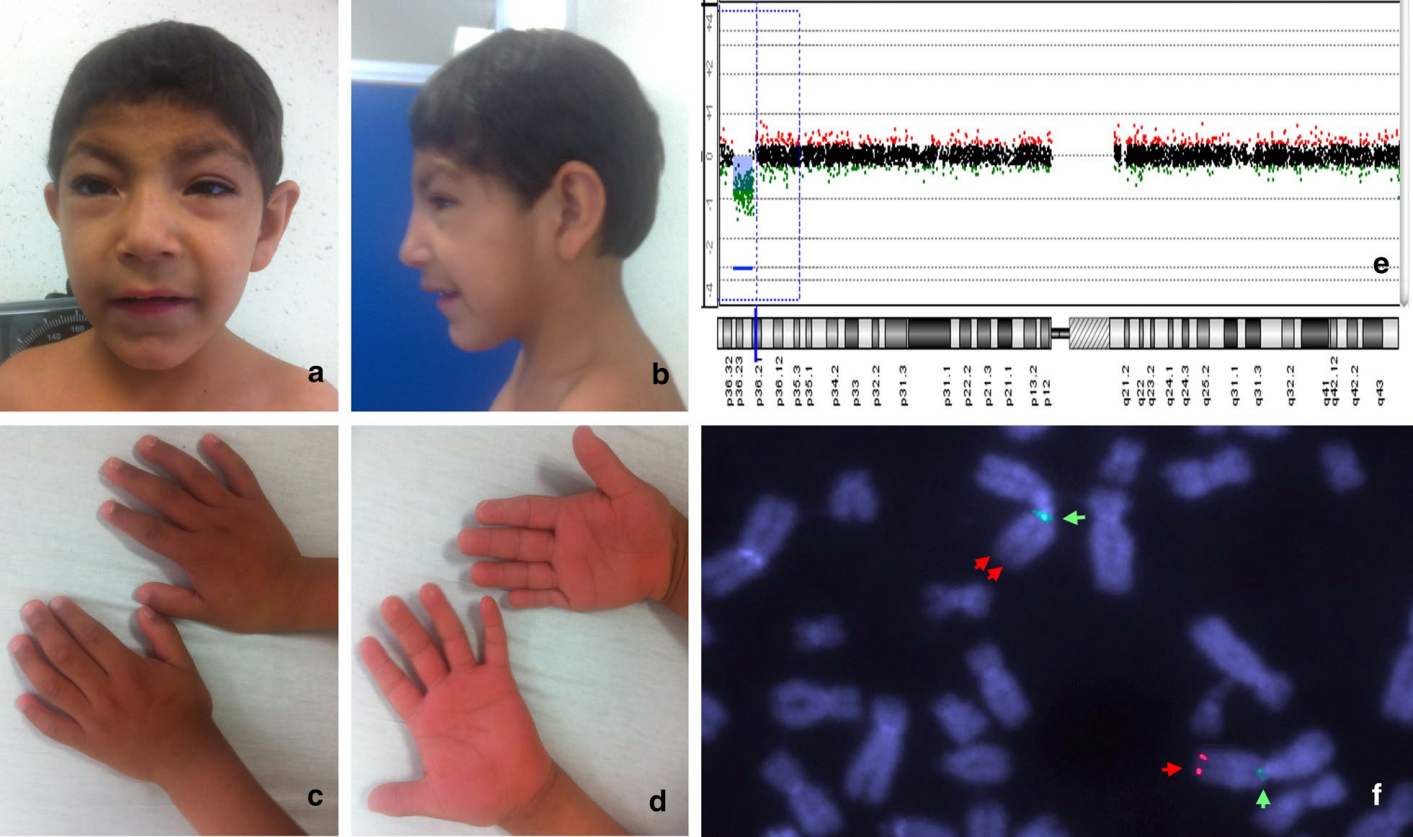
**a**–**d** Patient with coarse facies, narrow forehead, telecanthus, epicanthus, convergent strabismus, synophrys, hirsutism, broad nasal bridge, large ears, brachydactyly, and aberrant palmar creases; **e** aCGH showing an interstitial deletion of 7.2 Mb with breakpoints in bands 1p36.31p36.21: arr[GRCh37/hg19] 1p36.31p36.21(5414227_12632782)x1 dn; **f** DAPI counterstain FISH for the 1p36 region on metaphase chromosomes: locus specific probe for 1p36 marked with red fluorochrome (SureFISH, Agilent technologies, Santa Clara, USA) (one red arrow) and alpha satellite probe for chromosome 1 (chr1 CEP probe) marked with green fluorochrome (SureFISH, Agilent technologies, Santa Clara, USA) (one green arrow); deleted chromosome 1 with only one green fluorescent signal (one green arrow) and missing the red signal (two red arrows). Analysis was performed using an AXIO ImagerMI (Zeiss, Germany) microscope, and the images were obtained and analyzed using ISIS software (Meta Systems, Germany)

The patient is a 13 years-old boy, the first child of healthy and non-consanguineous parents. He was born after an uneventful pregnancy; his birth weight was 3075 g (> 10th percentile), and his length was 52 cm (75th percentile). At 6 months of age, the patient showed significant developmental delay and hypotonia. He was evaluated by the Genetics Department when he was 2 years old and he was able to speak, follow simple orders, identify colors, and count from 1 to 5; at 5 years old he had bladder and bowel control, however, he continued to display developmental delay, and currently goes to a special school, only speaks 10 words, and only counts to 10. On physical examination, he showed weight, height, and cranial circumference below the 3rd percentile; coarse facies; narrow forehead; telecanthus; epicanthus; convergent strabismus; synophrys; hirsutism; a broad nasal bridge; large ears; teletelia; diastasis recti; retractile testes; and hands with brachydactyly and aberrant palmar creases (Fig. 1a–d). His ophthalmologic exam found limitations to abduction (right eye − 4; left eye − 3) with palpebral retraction and shots, as well as hypermetropia of the right eye, concluding the diagnosis of bilateral DRS type 1. The prognosis of the patient is favorable, even until today he has had good evolution. His condition has not required any type of surgical intervention or specific treatment and consequently, he has not presented adverse events, anticipated events, or therapeutic changes.

**Fig. 2:**
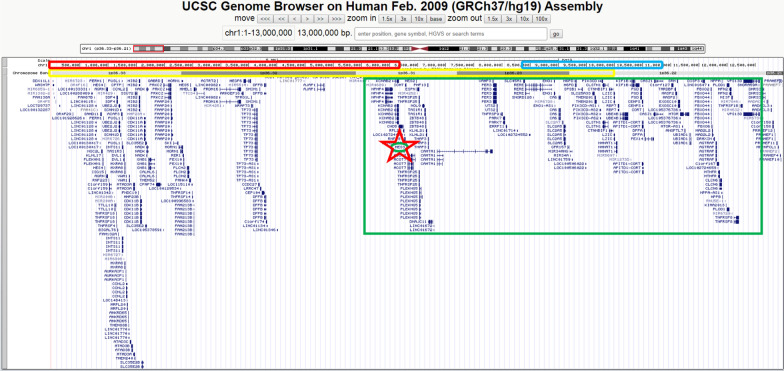
The USCS genome browser (GRCh37/hg19) showed more than 80 genes involved in the deleted region in our patient (green rectangle) which overlaps with both the proximal region (red rectangle) and the distal region (blue rectangle). Also, we showed that the *HES3* gene (red star) is included in both our patient’s deleted region (green rectangle) and Neal’s deleted region (yellow rectangle)

G-banded karyotype, echocardiogram, and cerebral computed axial tomography scans did not reveal any alteration; aCGH showed an interstitial deletion of 7.2 Mb with breakpoints at bands 1p36.31p36.21 (5,414,227–12,632,782; Fig. 1e). We confirmed this result using FISH analysis (Fig. 1f). The patient’s karyotype was 46,XY.ish del(1)(p36.31p36.21)(RPL22-).arr[GRCh37/hg19] 1p36.31p36.21(5414227_12632782)x1 dn. (ISCN 2016) [9]. The deletion encompassed more than 80 genes (USCS genome browser, GRCh37/hg19; Fig. 2). Both parents had normal karyotype, aCGH, and FISH analyses (Data not shown).

## Discussion

Although 1p36 deletion syndrome is clinically recognizable, there is significant phenotypic variation among affected individuals [10, 11]. This variation could be due to its genetic heterogeneity, which includes terminal and interstitial deletions of different lengths located throughout the 1p36 region; Wu et al. [12] proposed that most genes contributing to the phenotypic features of 1p36 deletion syndrome were located distal to marker D1S2870 in 1p36.31 (chr1: 6,289,764–6,289,973), and subsequently this region was referred as the distal or classical region. Later, a further detailed analysis of this distal region was published by Shimada et al., defining a narrow critical region located at 1.8 to 2.2 Mb for the classical phenotype, and another region at 5.4 to 6.2 Mb associated with a more severe intellectual disability [13]. On the other hand, Kang et al. [5] identified interstitial deletions affecting 1p36.23–1p36.11 in five individuals using aCGH, concluding that the features seen in these children might constitute a distinct proximal 1p36 deletion syndrome located at 1p36.2 (chr1: 8,395,179–11,362,893; Fig. 2). Our patient’s deletion overlaps distal and proximal regions, but not the narrow critical region for the classical phenotype (Fig. 2).

Clinically, our patient shared the developmental delay, generalized hypotonia, and cryptorchidism with the classical phenotype, but did not share the typical facial dysmorphism; he also shared the coarse facies, hirsutism, and the marked intellectual disability with the proximal phenotype. In addition, he displayed DRS, a congenital eye movement disorder characterized by a limited horizontal gaze and retraction of the globe into the orbit on attempted abduction, resulting in secondary narrowing of the palpebral fissure [14–16].

In 1974, Huber described three types of DRS. Type 1 is characterized by the marked limitation of abduction with normal or minimally defective adduction, while type 2 has normal or minimally defective abduction but marked limitation of adduction, and type 3 is characterized by the marked limitation of both abduction and adduction [7, 8, 17, 18]. In 2005, Kim and Hwang analyzed whether the presence of the abducens nerve changed depending upon the type of DRS, and found that the abducens nerve on the affected side was absent in all patients with type 1 DRS and in some type 3 DRS, but present in all patients with DRS type 2 [19].

Isolated DRS is usually sporadic, and less than 10% of cases show a familial pattern with autosomal dominant inheritance [7, 8, 16]. Some families with incomplete penetrance (the disease skips a generation) and variable expressivity (ranges in severity) have been documented [8]. DRS has a prevalence of about 1/1,000 in the general population, with no particular race or ethnic group showing a predisposition but shows up to 60% predominance among females [7, 8]. Prior reviews of DRS reported that this condition represents a 1–4% proportion of all strabismus cases, and that it is mostly unilateral and left-sided [8]. Electromyographic and MRI studies of patients with isolated DRS, revealed the absence of the abducens nerve and nuclei (6th cranial nerve) from the brainstem, and lateral rectus muscles partially innervated by the branches from the oculomotor nerves (3th cranial nerve) [8, 15, 20]. In accordance, it is currently accepted that DRS derives from developmental errors in the innervation of the ocular and facial muscles and it is included as one of the Congenital Cranial Dysinnervation Disorders [21–23].

### Association between 1p36 microdeletion and DRS

Large series, such as those of Battaglia et al. [1] or Shapira et al. [10], have reported ocular manifestations in 52–75% of patients with 1p36 microdeletion, including strabismus in 30–35% [1, 2], but none of them mentioned DRS; in fact, none of the genes responsible for the clinical manifestations of classical 1p36 microdeletion have been associated with DRS (Table 1) [13, 24, 25].

**Table 1:** Main candidate genes in the proximal and distal regions that contribute to the common clinical manifestations of 1p36 deletion syndrome

Gene	Function	Related phenotypes	Gen location (critical region)	References
MMP23B^a^	Metallopeptidase that is involved in bone matrix resorption and bone remodeling. Expressed in the cranial sutures	Large, late-closing anterior fontanel; Craniosynostosis (in duplications)	Chr1: 1,567,560–1,570,030 (Distal Region)	[31, 32]
GABRD^a^	Subunit of a pentameric ligand-gated chloride channel that is activated by GABA	Neurodevelopmental abnormalities, neuropsychiatric problems, seizures	Chr1: 1,950,768–1,962,192 (Distal Region)	[31]
SKI^a^	Protein that acts as a transcriptional co-regulator. Involved in neural tube development and muscle differentiation	Developmental delay, intellectual disability, seizures, orofacial clefting, congenital heart defects	Chr1: 2,160,134–2,241,652 (Distal Region)	[15, 31, 32]
PRDM16^a^	Zinc finger transcription factor. Interacts physically with SKI to inhibit transforming growth factor- β signaling	Left ventricular non-compaction, dilated cardiomyopathy	Chr1: 2,985,742–3,355,185 (Distal Region)	[31]
KCNAB2^a,b^	Auxiliary protein that alters the properties of functional potassium voltage-gated alpha subunits which are implicated in regulating neurotransmitter release, heart rate, neuronal excitability, smooth muscle contraction and cell volume	Developmental delay, intellectual disability, seizures	Chr1: 6,052,358–6,161,253 (Distal Region)	[31, 32]
CHD5^a,b^	Tumor suppressor gene. Encodes a neuron-specific protein, involved in chromatin remodeling and gene transcription, regulating the expression of neuronal genes	Intellectual disability Neuroblastoma	Chr1: 6,161,847–6,240,194 (Distal Region)	[5, 15, 31]
HES3^b^	Hes family bHLH transcription factor 3 implicated in the oculomotor nerve development	Oculomotor alterations, Duane Retraction Syndrome, probably	Chr1: 6,244,179–6,245,578 (Between Distal and Proximal Regions)	[27]
RERE^b^	Widely expressed nuclear receptor co-regulator. Reported to play a critical role in early cardiovascular development	Short stature, developmental delay, intellectual disability, brain anomalies, vision problems, hearing loss, renal anomalies, congenital heart defects, cardiomyopathy	Chr1: 8,412,464–8,877,699 (Between Distal and Proximal Region)	[15, 31]
UBE4B^b,c^	Ubiquitination factor that is involved in multiubiquitin chain assembly	Cardiomyopathy and neurodevelopmental phenotypes	Chr1: 10,093,041–10,241,297 (Proximal Region)	[31]
CASZ1^b,c^	Zinc finger transcription factor that is highly expressed in the heart	Congenital heart defects and cardiomyopathy	Chr1: 10,696,666–10,856,733 (Proximal Region)	[31]
PDPN	Integral membrane glycoprotein, which is preferentially expressed in the vascular endothelium	Congenital heart defects, cardiomyopathy	Chr1: 13,910,252–13,944,452 (Out of proximal region)	[31]
SPEN	Transcriptional repressor that may function as a nuclear matrix platform that organizes and integrates transcriptional responses	Congenital heart defects, cardiomyopathy, short stature, neurodevelopmental phenotypes	Chr1: 16,174,359–16,266,950 (Out of proximal region)	[31]
ECEI	Metalloprotease that is involved in the proteolytic processing of endothelin precursors to biologically active peptides	Congenital heart defects	Chr1: 21,543,740–21,672,034 (Out of proximal region)	[31]
HSPG2	Large multidomain heparan sulfate proteoglycan of the extracellular matrix that binds to various basement membrane proteins	Cleft palate, congenital heart defects	Chr1: 22,148,737–22,263,750 (Out of proximal region)	[31]
LUZPI	Leucine zipper protein 1 gene	Congenital heart defects, cleft palate, brain anomalies	Chr1: 23,410,516–23,495,3518 (Out of proximal region)	[31]

^a^Genes involved distal or classical 1p36 deletion phenotype. ^b^Genes deleted in our patient. ^c^Genes involved in the proximal 1p36 deletion

Neal et al. reported a patient with 1p36 microdeletion and periventricular nodular heterotopia who also displayed DRS [6]. The patient had a terminal deletion of 9.6 Mb, sharing with our patient the 1p36.22 region; the authors did not propose any candidate gene for DRS. In a detailed review of all the genes located in the overlapped region of Neal’s patient and our patient (Fig. 2), we found that the *HES3* gene could correlate with the pathophysiology of DRS, since this gene has been associated with the morphogenesis of the midbrain-hindbrain boundary and anterior hindbrain, and the development of the oculomotor nerve (3rd cranial nerve) [26–30].

*HES* family genes are the mammalian homologues of the Hairy and Enhancer Split genes in *Drosophila*, required for normal neurogenesis; they encode basic helix–loop–helix (HLH) transcriptional repressors [28–30]. In human, there are seven members in the *HES* family, *HES1*–*7*, which regulate developmental pathways in several tissues, including brain morphogenesis [29]. The developing nervous system is divided into many compartments by boundary structures; several knockout mice studies demonstrated that *Hes* genes are crucial to maintaining these boundary structures in the developing brain [27, 28]. The embryonic hindbrain is divisible into eight rhombomeres (Rh) [31], the abducens motor nucleus is included in Rh6 which is visible at stage 16 of human embryo development (before 6-week), when all the neuromeres have appeared [32, 33]. In this way, the haploinsufficiency of the *HES3* gene could be related to the altered ocular mobility, specifically DRS in our patient, by affecting the nerves and nuclei of both the 3rd cranial nerve derived from the midbrain close to the hindbrain junction and the 6th cranial nerve derived partially from Rh6.

We looked for other previously reported patients who had 1p36 deletions that included the *HES3* gene and compared them with our patient (Table 2). In addition to Neal’s patient [6], Shimada et al. analyzed 50 patients with different levels of 1p36 deletion, 18 of which presented loss of the *HES3*-containing region (Fig. 2), yet only one (patient 46) was reported to have an oculomotor disturbance; however, among the 17 remaining patients, four had strabismus [24]. It could be possible that some of the cases reported as strabismus may in fact be DRS, although the globe retraction and the narrowing of the eyelid fissure are visible data on physical examination, but if the extraocular motility test was not performed, DRS might not have been detected. Even so, only a part of the patients with *HES3* deletion present eye disorders; the absence of DRS in these patients may be related with incomplete penetrance and variable expressivity, already observed in the familial type of DRS [34–36]. It is then necessary to study other patients with del(1p36) with haploinsufficiency of *HES3,* to explore its role in the presence of DRS type 1.

**Table 2:** Description of deleted genomic regions of patients with 1p36 monosomy and Duane retraction syndrome

Patients	Chromosomal Region	Initial nucleotide	Final nucleotide	DRS
Present patient	1p36.31p36.21	5,414,227	12,632,782	Yes
Neal et al. [6]	1pter–p36.22	1	9,600,000	Yes
Shimada et al. [15] (Patient 46)	1pter–p36.22	1	10,001,011	Possible^a^

*HES3* gene located in chr1: 6,244,179–6,245,578

*DRS* Duane retraction syndrome

^a^Oculomotor disturbance; Distal critical region (classical) 1pter–p36.31 = 1–6,289,973 [13, 14]; Proximal critical region 1p36.23–p36.22 = 8,395,179–11,362,893 [5, 14]

Furthermore, non-syndromic DRS can be due to mutations in genes other than *HES3*, DRS1 in 8q13—OMIM#126800; DRS2 (*CHN1* gene in 2q31.1)—OMIM#118423; DRS3 (*MAFB* gene in 20q12)—OMIM#608968, which have an autosomal dominant inheritance. Although no other cases of DRS were found in the parents or other relatives of the present index case, and that does not support an additional mutation outside of 1p36, not having intentionally searched for these mutations is a limitation of the present study.

In conclusion, we propose that in non-classic 1p36 deletion syndrome, the *HES3* gene could be associated with oculomotor alterations, including DRS. However, further studies and more patients that include a complete clinical history and physical examination, as well as molecular description, are needed to confirm this finding. Finally, because DRS could be mistaken for common strabismus if it is not intentionally sought, we recommend a detailed ophthalmologic evaluation in all patients with 1p36 deletion.

## Data Availability

Routine ophthalmologic examination included best corrected visual acuity (BCVA), slit lamp examination, Goldmann applanation tonometry, gonioscopy, and dilated fundus examination. Genomic DNA from the patient and both parents was amplified and labeled using the CGH-Labeling Kit for Oligo Arrays (Enzo Life Sciences, USA), and was then applied to 60 k oligonucleotide arrays according to the manufacturer´s protocol (Agilent, Santa Clara, USA). The aCGH was performed using paired-samples, first pairing the patient and mother and then pairing the patient and father. Slides were scanned using a microarray scanner with Surescan High Resolution Technology (Agilent, Santa Clara, USA). Image quantification, array quality control, and aberration detection were performed using Agilent Feature Extraction and DNA Analytics software (Agilent, Santa Clara, USA) according to the manufacturer’s instructions. The deletion was verified using SureFISH probes (Agilent). Changes identified in the samples were visualized using the UCSC Genome Browser website (http://genome.ucsc.edu) and compared to the Database of Genomic Variants (http://projects.tcag.ca/variation) to exclude benign variants. The DECIPHER (https://decipher.sanger.ac.uk/) and ECARUCA (http://umcecaruca01.extern.umcn.nl:8080/ecaruca/ecaruca.jsp) databases were used as resources to aid in genotype–phenotype correlation.
